# ACRNaCT trial protocol: efficacy of adjuvant chemotherapy in patients with clinical T3b/T4, N+ rectal Cancer undergoing Neoadjuvant Chemoradiotherapy: a pathology-oriented, prospective, multicenter, randomized, open-label, parallel group clinical trial

**DOI:** 10.1186/s12885-019-6289-6

**Published:** 2019-11-15

**Authors:** Qingguo Li, Dakui Luo, Ji Zhu, Lifeng Yang, Qi Liu, Yanlei Ma, Lei Liang, Sanjun Cai, Zhen Zhang, Xinxiang Li, ACRNaCT study group

**Affiliations:** 1 Department of Colorectal Surgery, Fudan University Shanghai Cancer Center, No. 270, Dong’an Road, Xuhui District, Shanghai, 200032 China; 2 Department of Radiation Oncology, Fudan University Shanghai Cancer Center, No. 270, Dong’an Road, Xuhui District, Shanghai, 200032 China; 3 Department of Oncology, Shanghai Medical College, Fudan University, No. 270, Dong’an Road, Xuhui District, Shanghai, 200032 China

**Keywords:** Neoadjuvant chemoradiotherapy, Adjuvant chemotherapy, Locally advanced rectal cancer, Yield pathological stage

## Abstract

**Background:**

The CAO/ARO/AIO-94 demonstrated that neoadjuvant chemoradiotherapy (CRT) could decrease the rate of local recurrence rather than distal metastases in advanced rectal cancer. Adjuvant chemotherapy (ACT) can eliminate micrometastasis, and render a better prognosis to rectal cancer. However, adoption of ACT mainly depends on the evidence from colon cancer. Neoadjuvant CRT can lead to tumor shrinkage in a number of patients with advanced rectal cancer. The administration of adjuvant therapy depending on pretreatment clinical stage or postoperative yield pathological (yp) stage remains controversial. At present, the clinical guidelines recommend ACT for patients with stage II/III (ypT3–4 N0 or ypTanyN1–2) rectal cancer following neoadjuvant CRT and surgery. However, the yp stage may influence the guidance of ACT.

**Methods:**

According to the postoperative pathological stage, the present study was divided into two parts with different study design procedures. Patients will undergo different therapeutic strategies after collecting data related to postoperative pathological stage. For patients with pathologic complete response or yp stage I, the study was designed as a non-inferiority trial to compare the patients’ long-term outcomes in observational group and those in treatment group with 5-fluorouracil. For patients at yp stage II or III, the study was designed as a superiority trial to compare the oncological effect of oxaliplatin combined with 5-fluorouracil, in addition to 5-fluorouracil alone in ACT. The primary endpoint is 3-year disease-free survival (DFS). Secondary endpoints are 3-year, 5-year overall survival, 5-year DFS, and the rate of local recurrence and adverse events resulted from chemotherapy and the patients’ quality of life postoperatively.

**Discussion:**

The ACRNaCT trial aims to investigate whether observation is not inferior than 5-fluorouracil for pathologic complete response or yp stage I, and indicate whether combined chemotherapy contains superior outcomes than 5-fluorouracil alone for yp stage II or III in patients receiving neoadjuvant CRT and surgery for locally advanced rectal cancer (LARC). This trial is expected to provide individualized adjuvant treatment strategies for LARC patients following neoadjuvant CRT and surgery.

**Trial registration:**

The trial has been registered in ClinicalTrials.gov on January 30, 2018 (Registration No. NCT03415763), and also, that was registered in Chinese Clinical Trial Registry on November 12, 2018 (Registration No. ChiCTR1800019445).

## Background

Colorectal cancer is the third most commonly occurring cancer in men and the second most commonly occurring cancer in women worldwide. A recent study conducted on 36 types of cancer in 185 countries demonstrated that rectal cancer is the eighth most frequently diagnosed malignancy and the tenth most common cause of cancer-related mortality [1]. A Surveillance, Epidemiology, and End Results population-based rectal cancer analysis found that 72.20% of all rectal cancer patients with evaluable TN category were categorized as locally advanced rectal cancer (LARC; T3/T4 N0, TxN+) [2].

The therapeutic strategy for LARC has evolved over the past decades. The adoption of the principles of total mesorectal excision (TME) has yielded satisfactory survival outcomes, as well as decreasing local recurrence rate of mid-low rectal cancer. TME is the excision of the tumor en bloc with its blood and lymphatic supply, that is, the mesorectum. However, local recurrence and distant metastases have been still major causes of cancer-related mortality after TME.

The results of a randomized German CAO/ARO/AIO trial on preoperative chemoradiotherapy (CRT) demonstrated a decreased local recurrence and therapeutic toxicity compared with postoperative CRT [3, 4]. Adjuvant chemotherapy (ACT) has a potential for removing micrometastasis, as well as improving overall survival (OS). ACT has been well applied in colon cancer patients with high-risk stage II and stage III disease. After CRT, a number of rectal cancer patients experienced downstaging of T or N. It is difficult to distinguish real ‘high-risk’ stage II and stage III disease from yield pathological stage. A systematic review reported that ACT resulted in a reduction in the risk of recurrence (25%) and mortality (17%) [5]. However, limitations of this study were obvious. Only two randomized controlled trials enrolled patients who received neoadjuvant CRT. Oxaliplatin was not used in ACT. Besides, the value of ACT was susceptible to adjuvant radiotherapy. The results of EORTC 22921 trial revealed that ACT after preoperative radiotherapy was not associated with disease-free survival (DFS) or OS [6]. Similarly, no survival benefit was observed in another three trials [7–9]. A systematic review and meta-analysis of data obtained from the above-mentioned four trials reached similar conclusions [10]. However, insufficient compliance to ACT is identified as a main challenge to evaluate the value of ACT in patients with rectal cancer after neoadjuvant CRT and surgery.

Findings from adjuvant oxaliplatin in rectal cancer (ADORE) trial demonstrated that adjuvant oxaliplatin, leucovorin, and 5-fluorouracil (FOLFOX) could improve DFS compared with fluorouracil plus leucovorin in patients with LARC after neoadjuvant CRT and surgery. A subgroup analysis revealed that the survival benefit was only observed in pathological stage III rather than pathological stage II [11]. Pathological stage might be a predictive factor for guiding the choice of ACT. Results of CAO/ARO/AIO-04 trial also showed that DFS improved in patients who received oxaliplatin plus fluorouracil compared with those who received only fluorouracil [12]. At present, clinical guidelines do not make a robust proposal in favor of adoption of ACT for LARC patients after neoadjuvant CRT and surgery.

After CRT, patients may experience different levels of downstaging due to tumor heterogeneity, and about 20% of patients presented pathologic complete response. Multiple studies have demonstrated that tumor regression grade (TRG) is an independent prognostic factor [13–15]. Nevertheless, no clear evidence exists for TRG as a predictive marker for administration of ACT. A retrospective study reported the survival outcomes of patients who received neoadjuvant CRT, and experienced a pathologic complete response with the help of observation alone [16]. The 5-year DFS and OS rates of these patients were 96 and 100%, respectively. Similarly, results from the Surveillance, Epidemiology, and End Results database demonstrated that ACT seemed not to have survival benefit for rectal cancer patients with yield pathological (yp) Tis-2 N0 [17]. Furthermore, a pooled analysis of 3313 patients demonstrated that patients with a pathological complete response (pCR) after CRT may not benefit from ACT, while patients with residual lesion had slightly superior prognosis in the adjuvant setting [18]. However, evidence from National Cancer Database of Canada indicated that ACT was associated with better overall survival in patients with locally advanced rectal cancer who achieve a pCR. Stratified analysis revealed that only those patients with pretreatment node-positive disease benefited from administration of ACT [19]. A systematic review and meta-analysis included in 6 studies based on 18 centres or databases involving 2948 rectal cancer patients with pCR, and indicated that ACT is associated with improved OS in locally advanced rectal cancer patients with pCR after neoadjuvant CRT and radical surgery [20]. From the inspiration of these retrospective studies, we attempted to perform ACRNaCT trial to address the value of ACT in LARC patients who received neoadjuvant CRT and surgery.

## Methods/design

### Study design and participants

**Fig. 1:**
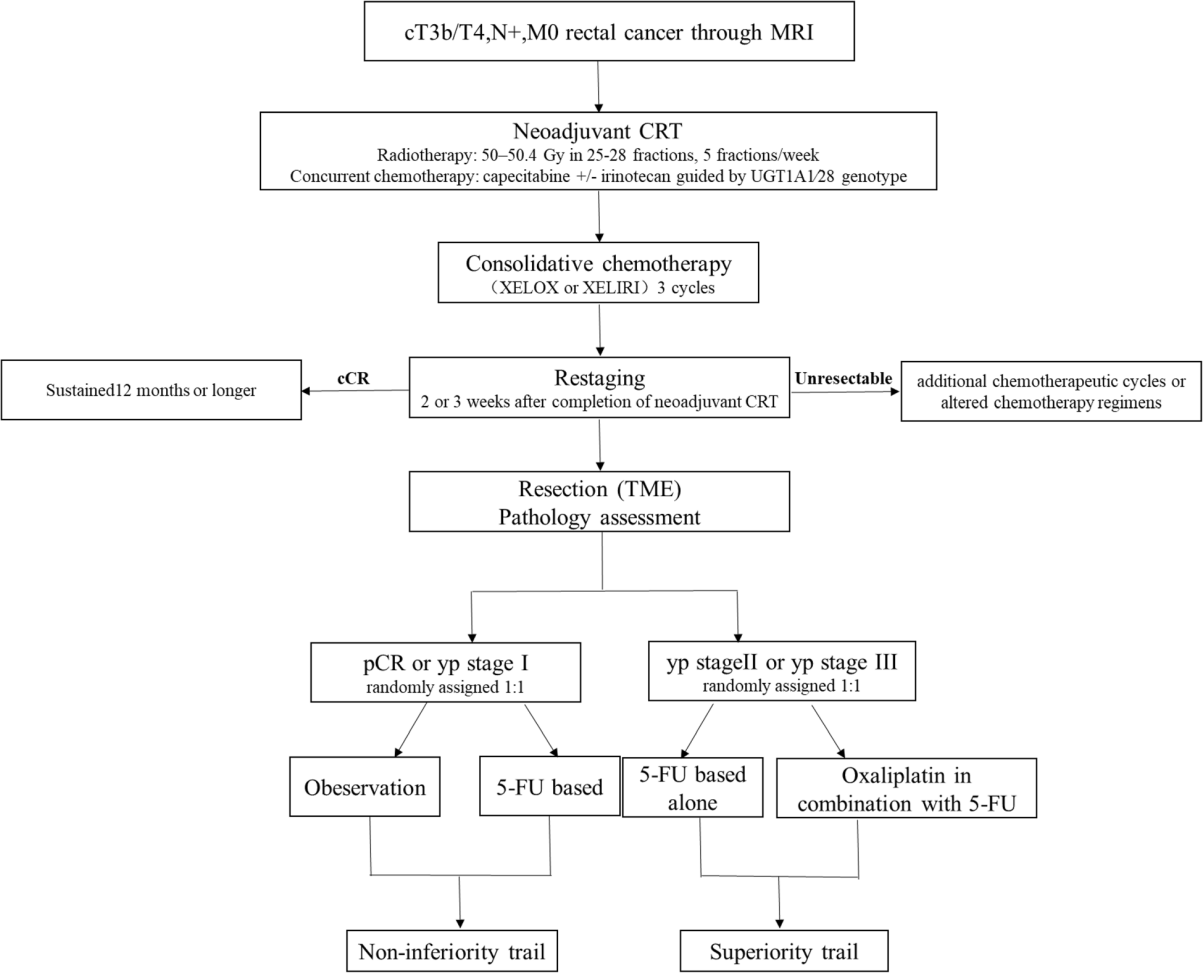
Overview of ACRNaCT trail

An Adjuvant chemotherapy in patients with clinical T3b/T4,N+ Rectal cancer undergoing Neoadjuvant Chemoradiotherapy (ACRNaCT) trial was conducted as a prospective, multicenter, randomized phase III study, consisting of two parts based on postoperative pathological stage. Overviews of enrollment and interventions are depicted in Fig. 1. Patients will be recruited from 29 Chinese institutions. Eligible patients will be diagnosed through magnetic resonance imaging (MRI) with T3b/T4,N+ rectal cancer and are histologically confirmed adenocarcinoma of the rectum (size of tumor < 10 cm from anal verge) without distant metastases. There will be no contraindications of anesthesia, operation, and CRT as well. Inclusion criteria include: (1) Age 18 to 75 years. (2) The lesion must be within 12 cm of the anus as measured by endoscopy. (3) Histologically confirmed diagnosis of rectal carcinoma. (4) Computed tomography (CT), MRI, and endoscopic ultrasound (EUS) verified as clinical stage III rectal cancer without involvement of other organs. (5) No evidence of multiple primary cancers. (6) Sufficient organ function. (7) Signed written informed consent form. Exclusion criteria include: (1) Younger than 18 or older than 75 years. (2) Synchronous or metachronous malignancy within 5 years. (3) Patients with intestinal obstruction, perforation or bleeding who require emergency surgery. (4) Patients with a history of pelvic irradiation. The American Society of Anesthesiologists (ASA) grade IV or V. (5) Women who are pregnant (confirmed by serum b-HCG in women of reproductive age) or breast feeding. (6) Severe mental disorders. (7) Patients with severe emphysema, interstitial pneumonia, or ischemic heart disease who cannot tolerate surgery. (8) Patients who received steroid therapy within one month. (9) Patients or family members misunderstand the conditions and goals of this study. The withdrawal criteria include: (1) Distant metastasis is confirmed by abdominal exploration or postoperative pathology. (2) Patients receive other treatment, which is not related to the present study. The situation that patients receive subsequent therapy after relapse or metastasis is permitted. (3) Patients with intestinal obstruction or perforation or bleeding who require emergency surgery after inclusion in the study. (4) Patients decide to withdraw from the study with any reasons. (5) Patients who cannot receive therapy any more due to non-neoplastic diseases. (6) Patients who cannot finish planed procedure due to any reasons.

For patients with pCR or yp stage I, the study was designed as a non-inferiority trial to compare the long-term outcomes of patients in observational group and those in treatment group with 5-fluorouracil. For patients with yp stage II or III, the study was designed as a superiority trial of 5-fluorouracil in combination with oxaliplatin compared with 5-fluorouracil alone in adjuvant setting. Participants will be allocated 1:1 to an intervention group or a control group after obtaining pathological data. Recruitment initiated in November 2018 and is expected to be completed in December 2020. The trial has been registered in ClinicalTrials.gov on January 30, 2018 (Registration No. NCT03415763), and also, that was registered in Chinese Clinical Trial Registry on November 12, 2018 (Registration No. ChiCTR1800019445).

### Sample size

The sample size is based on the log-rank test. For patients with pCR or yp stage I, the estimated 3-year DFS was 85% for the 5-fluorouracil and 75% for the observation arm. With 80% power and probability errors of 5%, the upper limit of the two-sided 95% confidence interval (95% CI) of the hazard ratio (HR) will not exceed 1.6. Almost 192 patients will be required in each study group. For patients with yp stage II or III, the estimated 3-year DFS was 55% for the 5-fluorouracil alone and 67% for 5-fluorouracil in combination with oxaliplatin arm. With 80% power and probability errors of 5%, approximately 190 patients will be required in each study group.

### Randomization process

The minimization technique will be used in the current trial. Stratification factors are age, yield pathological stage and distance from tumor to the anal verge. Patients will be randomly assigned using a central randomization system without masking.

### Neoadjuvant radiotherapy

Preoperative radiotherapy will be consisted of 50–50.4 Gy in 25–28 fractions (1.8–2 Gy), 5 fractions/week. Intensity-modulated radiation therapy will be conducted for the whole pelvis.

### Concurrent chemotherapy

Capecitabine is administrated concurrently with radiotherapy from day 1 to day 5. Patients receive capecitabine (825 mg/m^2^) orally. Alternatively, irinotecan and capecitabine are delivered concurrently with radiotherapy. Patients receive capecitabine (625 mg/m^2^) orally in combination with weekly irinotecan for five consecutive weeks according to the UGT1A1/28 genotype (days 1, 8, 15, 22, and 29). The dose of weekly irinotecan is 80 mg/m^2^ in patients with the *1*1 genotype and 65 mg/m^2^ in those with the *1*28 genotype.

### Consolidative chemotherapy

Consolidative chemotherapy in form of XELOX (capecitabine and oxaliplatin) or XELIRI (capecitabine and irinotecan) is performed for three cycles for 3 weeks after completion of chemoradiotherapy. XELOX (oxaliplatin (130 mg/m^2^) as a 2-h infusion on day 1, followed by capecitabine (1000 mg/m^2^) twice daily for 14 days every 3 weeks) is delivered if patients receive capecitabine. XELIRI (irinotecan (200 mg/m^2^) on day 1, followed by capecitabine (1000 mg/m^2^) twice daily for 14 days every 3 weeks) is delivered if patients receive capecitabine in combination with weekly irinotecan.

### Resection

Patients will undergo restaging 2 or 3 weeks after completion of neoadjuvant CRT. Surgery will be undertaken following principles of TME for patients who are appropriate for undergoing resection. For patients who have locally unresectable disease, additional chemotherapeutic cycles are needed. Alternatively, chemotherapy regimens can be changed. Watch-and-wait nonoperative approach is not recommended for clinical complete responders in terms of high-risk of recurrence. For patients who refuse undergoing surgery, the risk of relapse will be informed. These participants will not be excluded from our study and sustained clinical complete response for 12 months or longer should be reached under intensive surveillance.

### Adjuvant chemotherapy

According to postoperative pathological stage, patients with pCR or yp stage I will be randomly assigned (1:1) into two groups of observation and ACT with 5-fluorouracil. Patients with yp stage II or III will also be randomly assigned to (1:1) the treatment groups receiving either 5-fluorouracil or 5-fluorouracil plus oxaliplatin as ACT. The use of a course of three cycles of ACT is recommended. In 5-fluorouracil group, patients daily receive capecitabine (1250 mg/m^2^) twice for 14 days every 3 weeks orally. In 5-fluorouracil plus oxaliplatin group, adoption of XELOX (oxaliplatin (130 mg/m^2^) as a 2-h infusion on day 1, followed by daily capecitabine (1000 mg/m^2^) twice for 14 days every 3 weeks) or mFOLFOX6 (oxaliplatin (85 mg/m^2^), leucovorin (400 mg/m^2^), and fluorouracil (400 mg/m^2^) followed by continuous infusion of fluorouracil (2400 mg/m^2^) for 46–48 h, repeated every 2 weeks) is optional.

### Follow-up

History, physical examination, tumor markers test, ultrasound imaging of abdomen and pelvis, and radiography of chest will be carried out every 3 months for the first 2 years and every 6 months thereafter. Abdominopelvic CT or MRI and CT scan of chest will be conducted annually. Colonoscopy will be scheduled at 1, 3, and 5 years for post-treatment surveillance.

### Outcomes

The primary endpoint is 3-year DFS and the secondary endpoints are 3- and 5-year OS, 5-year DFS, and the rate of local recurrence and adverse events resulted from CRT and patients’ quality of life. DFS is calculated from the date of surgery to the date of recurrence. OS is defined as the time from surgery to death.

### Data collection, management and monitoring

All data will be collected in form of an electronic case report form (eCRF) through an electronic data capture (EDC) system. The data center located at Fudan University Shanghai Cancer Center will be in charge for quality control of study data. The EDC system will check the data automatically and data managers will review the eCRFs regularly. The queries will be sent out to each investigator. The data monitoring committee (DMC) is made up of three surgeons who have no conflict of interest in this study. DMC is responsible for reviewing efficacy and toxicity of intervention independently from the investigators and reporting severe adverse events. No regular auditing is scheduled.

### Statistical analysis

Continuous measures will be compared using the Wilcoxon rank-sum test. Chi-square or Fisher’s exact test will be utilized to compare categorical variables. The DFS and OS will be estimated using the Kaplan-Meier method. The Cox proportional hazards model will be employed to identify the prognostic factors. No interim analysis will be planned as well.

### Protocol version

Version 1.0.

## Discussion

ADORE and the German CAO/ARO/AIO-04 trials demonstrated that FOLFOX as adjuvant regimen for LARC patients, receiving CRT and surgery associates with superior DFS compared with 5-FU alone [11, 12]. However, it is obviously unreasonable to adopt the same adjuvant regimen for patients with a pathologic complete response or yp stage III. In clinical practice, clinicians tend to recommend 5-FU alone for patients with a pathologic complete response and prefer to assign patients to combined chemotherapy, especially for patients with minimal residual disease. Nevertheless, high-level evidence is lacking. TRG is a well-established prognostic factor rather than a predictive marker for guiding the administration of ACT.

The ACRNaCT trial is the first pathology-oriented, prospective, randomized study, evaluating the value of ACT in patients with rectal cancer after neoadjuvant CRT and surgery. After the pathological assessment, the participants with pathologic complete response or yp stage I are randomized to either the intervention group, receiving 5-fluorouracil or control group, undergoing standard surveillance. The therapeutic efficiency may ultimately lead to reduce side effects of chemotherapy and patients’ financial burden without compromising oncological safety. The participants with yp stage II or III are randomized to either the monotherapy group, receiving 5-fluorouracil alone or the combined chemotherapy group, receiving 5-fluorouracil plus oxaliplatin. Evidence from ADORE and the German CAO/ARO/AIO-04 trials indicated that addition of oxaliplatin was superior than using 5-fluorouracil alone [11, 12]. The mentioned treatment strategy requires further verification in our trial for patients with poor TRG.

This is the first large randomized controlled trial on the value of ACT for advanced rectal cancer patients, receiving CRT and surgery based on postoperative pathological stage. This is of great significance that ACRNaCT will provide novel and individualized adjuvant treatment strategies for rectal cancer patients following neoadjuvant CRT and surgery.

## Data Availability

The datasets used and/or analysed during the current study are available from the corresponding author on reasonable request.

## Abbreviations

5-FU: 5-fluorouracil
cCR: clinical complete response
CRT: Chemoradiotherapy
DFS: Disease-free survival
LARC: Locally advanced rectal cancer
OS: Overall survival
pCR: pathologic complete response
TME: Total mesorectal excision
TRG: Tumor regression grade
yp: yield pathological
